# Synthesis of Minerals with Iron Oxide and Hydroxide Contents as a Sorption Medium to Remove Arsenic from Water for Human Consumption

**DOI:** 10.3390/ijerph13010069

**Published:** 2015-12-23

**Authors:** Sofia Garrido-Hoyos, Lourdes Romero-Velazquez

**Affiliations:** 1 Instituto Mexicano de Tecnología del Agua, Paseo Cuahnáhuac 8532, Col. Progreso, Jiutepec, CP. 62550 Morelos, México; 2 Universidad Politécnica del Estado de Morelos, Paseo Cuauhnáhuac 566, Col. Lomas del Texcal, Jiutepec, CP. 62550 Morelos, México; ambiental.romero@gmail.com

**Keywords:** arsenic, kinetic, experimental design, isotherm, adsorbent media

## Abstract

Arsenic has been classified as a toxic and carcinogenic chemical element. It therefore presents a serious environmental problem in different regions of the country and the world. In the present work, two adsorbent media were developed and evaluated to remove arsenic from water in the Pájaro Verde mine shaft, Huautla, Tlaquiltenango, Morelos. The media were synthesized and characterized, obtaining a surface area of 43.04 m^2^·g^−1^ for the goethite and 2.44 m^2^·g^−1^ for silica sand coated with Fe(III). To conduct the sorption kinetics and isotherms, a 2^3^ factorial design was performed for each medium in order to obtain the optimal conditions for the factors of arsenic concentration, pH and mass of the adsorbent. The best results were obtained for goethite, with a removal efficiency of 98.61% (C_0_ of As(V) 0.360 mg·L^−1^), and an effluent concentration of 0.005 mg·L^−1^, a value that complies with the modified Official Mexican Standard NOM-127-SSA1-1994 [1] and WHO guidelines (2004) [2]. The kinetic equation that best fit the experimental data was the pseudo-second-order, resulting in the highest values for the constants for synthetic goethite, with a rate constant sorption of 4.019·g·mg^−1^·min^−1^. With respect to the sorption isotherms, both media were fitted to the Langmuir-II linear model with a sorption capacity (q_m_) of 0.4822 mg·g^−1^ for goethite and 0.2494 mg·g^−1^ for silica sand coated with Fe(III).

## 1. Introduction

The presence of arsenic in water results from the dissolution of minerals, geogenic activities, industrial effluents and the air. Nevertheless, other causes of arsenic in nature depend on anthropogenic activities such as the leaching of mine residues [3] and the use of insecticides. The degree of the toxicity of arsenic depends on the way in which it dissolves in the medium in which it is found. Arsenic is present in its four forms of oxidation, as arsenate (As^5+^), arsenite (As^3+^), arsine (As^3−^) and its fundamental state (As^0^). Generally, the form in which it is present in water bodies is its trivalent and pentavalent state, with As^3+^ being the most toxic [4].

In Mexico, several cases of groundwater contamination have presented themselves, one of which is located in the Huautla in the municipality of Tlaquiltenango, State of Morelos. Another case of contamination is present in the Comarca Lagoon, with values as high as 0.348 mg·L^−1^, 14 times greater than the maximum limit permissible by the Mexican Standard NOM-127-SSA1-1994 and the WHO. In both of these locations, the water is frequently used as a source of supply for human consumption.

The consumption of water contaminated with arsenic can produce cancerous diseases in a variety of organs in the body, including the lungs, kidneys, bladder, liver and skin, as well as hydroarsenicism [5]. The aim of this work was to develop and evaluate the efficiency of two different sorption media, characterized by their iron oxides and hydroxides contents, for the removal of As(V) from water for human consumption.

## 2. Experimental Section

### 2.1. Preparation and Characterization of Adsorbent Media

Two adsorbent media were developed, silica sand coated with Fe(III) and synthetic goethite, following the methods by Thirunavukkrasu *et al.* (2001) [6] and Garrido (2008) [7], respectively. The media were analyzed in a Micrometrics Model ASAP202 surface area and porosity analyzer. The size of the particulate was determined by granulometry method. The adsorbent materials were characterized by analysis of elemental composition with energy dispersive X-ray spectroscopy (EDXS), surface area and porosity using a Micrometrics Model ASAP 2020 (No Series: 831Norcross, GA, USA); the morphology of this media was analyzed by scanning electron microscopy (SEM) (Quanta 200, Hillsboro, OR, USA), with secondary electrons (SE) and back-scattered electrons detector (BSE), which determined chemical changes in the contrast images. Functional groups on the media, responsible for the sorption of arsenic, were determined using Fourier transform infrared spectroscopy (FTIR) (Thermo Scientific, Madison, WI, USA), Nicolet 6700 spectrometry brand with detector: DTGS KB.

### 2.2. Batch Test

The water used for the batch tests was obtained from the Pájaro Verde mine shaft, in Huautla, the municipality of Tlaquiltenango, Morelos (Table 1). It was characterized using Mexican Standards and Standard Methods (2013) [8].

**Table 1:** Huautla mine shaft water quality.

Parameters	Units	Value
**Physical**		
Total dissolved solids	mg·L^−1^	226
Electrical conductivity	μS·cm^−1^	452
Turbidity	UTN	19
True color	UPt-Co	2
**Chemical**		
Total hardness	mg·L^−1^ CaCO_3_	179.9
pH		8.57
Redox potential	mV	233
Bicarbonates	mg·L^−1^	221
Chlorides	mg·L^−1^	2.4
O-Phosphates	mg·L^−1^	0.45
Nitrates	mg·L^−1^	6.3
Sulphates	mg·L^−1^	14
Arsenic	mg·L^−1^	0.196
Calcium	mg·L^−1^	35.49
Magnesium	mg·L^−1^	3.06

Batch adsorption kinetics was performed in a Jar Tester Phillips & Birds with agitation at 120–200 rpm at 25 °C [9]. The As(V) concentrations were prepared with sodium arsenate heptahydrate (Na_2_HAsO_4_·7H_2_O, 98% purity, (Sigma-Aldrich: Milwaukee, WI, USA), to reached the different values of the experimental design. The pH was maintained by adjusting with NaOH. Between 1 and 2 L of water was taken from Huautla mine shaft, to which were added different media masses, between 1.0 and 4 g. Samples were taken at time intervals of 25 min. The sample was filtered using a 0.45 μm membrane and the parameters arsenic and pH were analyzed. Arsenic was determined using the Wagtech photometer method [10,11]. The experiments were carried out using a 2^3^ factorial design for each medium with the program Statgraphics Centurion XVI, version 2012, obtaining a matrix containing 10 experimental tests. The factors evaluated were arsenic concentration, pH and mass of the adsorbent medium (Table 2).

**Table 2:** Experimental design for the batch test.

Factors	Silica Sand Coated with Fe(III)			Goethite		
	+1	0	−1	+1	0	−1
As concentration (mg·L^−1^)	0.392	0.294	0.196	0.360	0.278	0.196
pH	8.5	8	7.5	8.5	8	7.5
Adsorbent mass (g)	4	3	2	2	1.5	1

The quantity of arsenic adsorbed was deduced from the initial concentration using the equation:

(1) $$q=\frac{V(C_{0}-C_{e})}{2}$$

where *q* is the measured sorption per unit weight of solid, *V* is the volume of the solution, *C*_0_ and *C_e_* are the initial and equilibrium concentrations of arsenic, respectively, and *M* is the dry weight of the biosorbent.

To calculate the kinetic sorption constants, the experimental data were fit to a pseudo-second-order equation. For isotherms, the data were adjusted to the Langmuir (I) and (II) and Freundlich models.

## 3. Results and Discussion

Table 3 shows the results of the analysis of the composition of silica sand coated with Fe(III) and goethite. The primary elements present in both media are carbon (C), oxygen (O) and iron (Fe) with the highest content. Aluminum and silica compounds were found as constituents of the silica sand.

**Table 3:** Elemental analysis of adsorbents.

Element	Percentage by Weight (%)	
	Silica Sand Coated with Fe(III)	Goethite
C	18.57	15.50
O	38.77	33.68
Al	0.63	-
Si	9.77	-
Fe	32.25	50.82

Table 4 shows the characterization of the adsorbent media used in the batch tests. The surface area of goethite was observed to be 17 times greater than that of silica sand coated with Fe(III); both media were classified as mesoporous. In previous studies, such as that of Garrido (2008) [7], the greatest amount of As(V) found in water in Huautla was species HASO_4_^2−^, according to the species diagram at pH 8.57 [9], with a radius of 3.97 Å. Therefore, the diameters of both media were porous enough for the As^5+^ molecule to enter.

**Table 4:** Characterization of the adsorbent media obtained in the laboratory.

Analysis	Units	Silica Sand Coated with Fe(III)	Goethite
Surface area	m^2^·g^−1^	2.44	43.04
Accumulated micropore volume	cm^3^·g^−1^	0.002	0.12
Average pore diameter	A	53.60	119.05
Pore structure		Mesoporous	Mesoporous
Particulate size	mm	0.3–0.6	0.1–0.6

**Figure 1:**
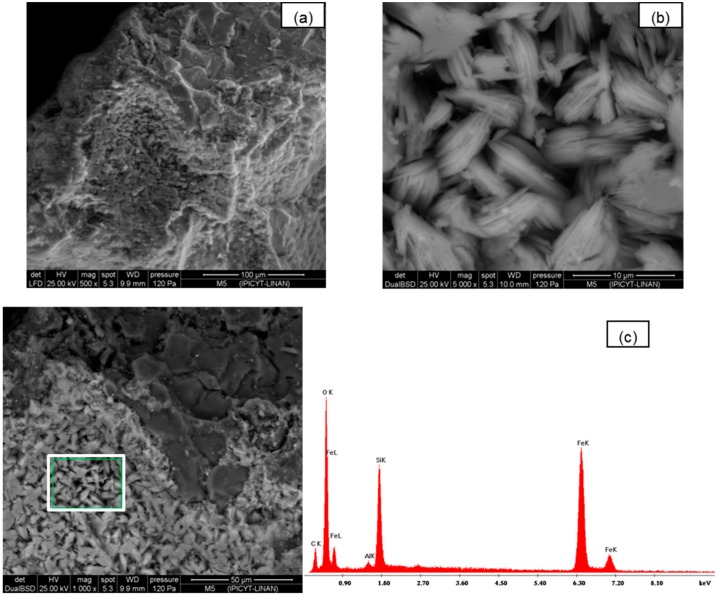
(**a**,**b**) SEM images of silica sand coated with Fe(III) untreated; (**c**) Fragment where EDS (white box) and chemical spectrograph quantification EDS of adsorbent was performed.

**Figure 2:**
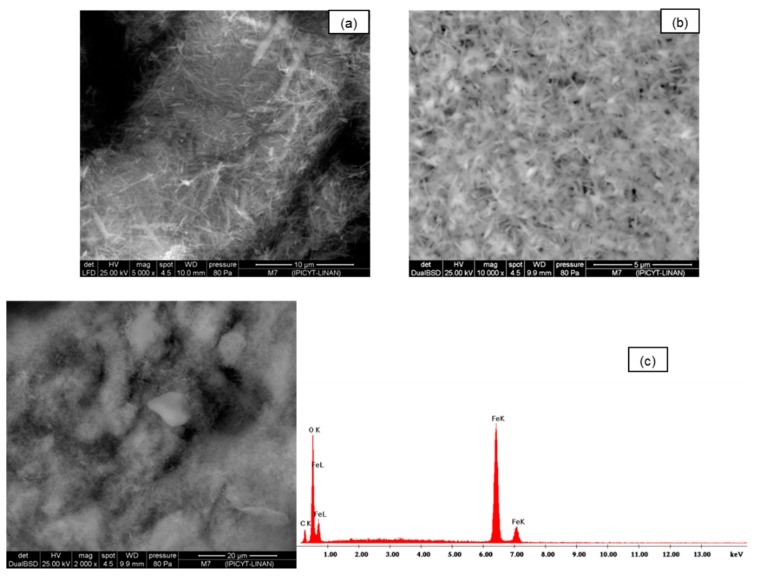
(**a**,**b**) SEM images of goethite untreated; (**c**) Chemical spectrograph quantification EDS of adsorbent was performed.

Scanning electron microscopy (SEM) was used to study the morphological structure of the two media. In Figure 1a–c, the images of silica sand coated with Fe(III) untreated are presented. In these images the surface amorphous structure is shown. In Figure 2a–c, the SEM images of synthetic goethite, which is a material of different sizes, are observed at 2000×; when this is viewed at higher magnifications of 5000× and 1000×, very fine fibers that are less than 5 μm with crystalline materials of spherical structure are shown.

**Figure 3:**
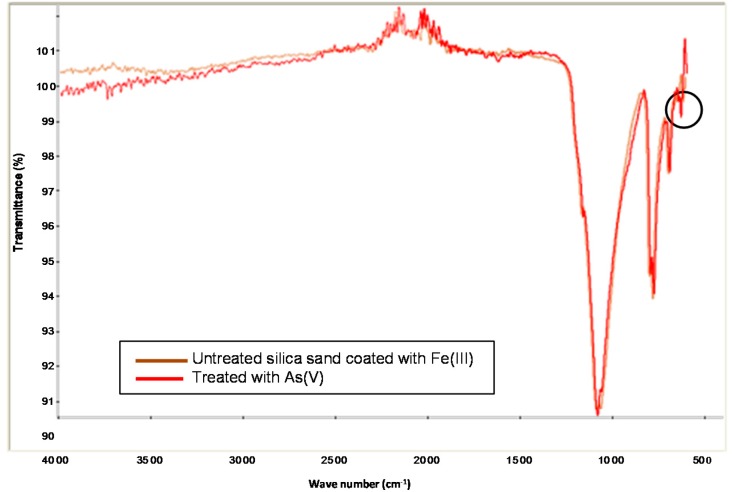
FTIR spectrum of silica sand coated with Fe(III) in absence and presence of As(V).

The infrared spectra (IR) from silica sand coated with Fe(III) in the absence and presence of As(V), as shown in Figure 3, were used to analyze the presence of the main sorption functional groups. It is noted that the two spectra are very similar. The small peak at 1635 cm^−1^ indicates the presence of free water molecules and water molecules bonded onto silica. The intense band at 1160 cm^−1^ is characteristic of Si-O bonds as is the peak noticeable at 800 cm^−1^. The band in the presence of As(V) between 600 and 520 cm^−1^ belongs to the stretching mode Fe-O, and Fe-O-As, small shoulder, respectively [12].

**Figure 4:**
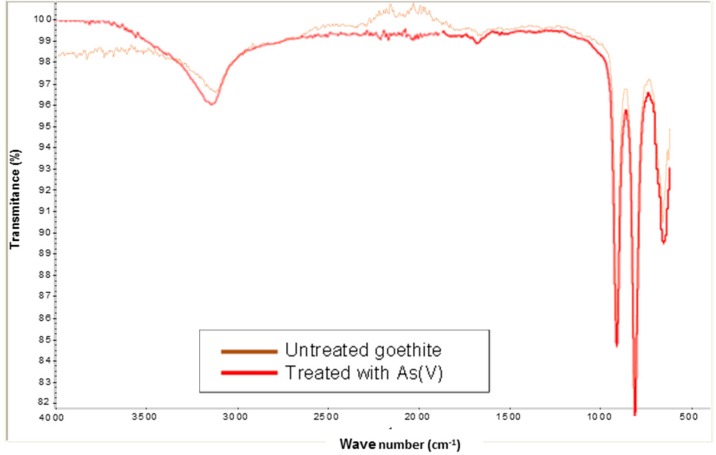
FTIR spectrum of goethite in absence and presence of As(V).

Figure 4 shows the spectrum (IR) from goethite in the absence and presence of As(V). It is noted that the two spectra are very similar. The region of 3500–3000 cm^−1^ reveals a very high increase in O-H stretching with for both bands. The band in the absence and presence of As(V) between 600 and 520 cm^−1^ belongs to the stretching mode Fe-O [12]. The band at 780–800 cm^−1^ observed in the presence of arsenic can be assigned to the As-OH stretching [12,13]. It may be due to the sorption of HAsO4^2−^ on goethite.

**Figure 5:**
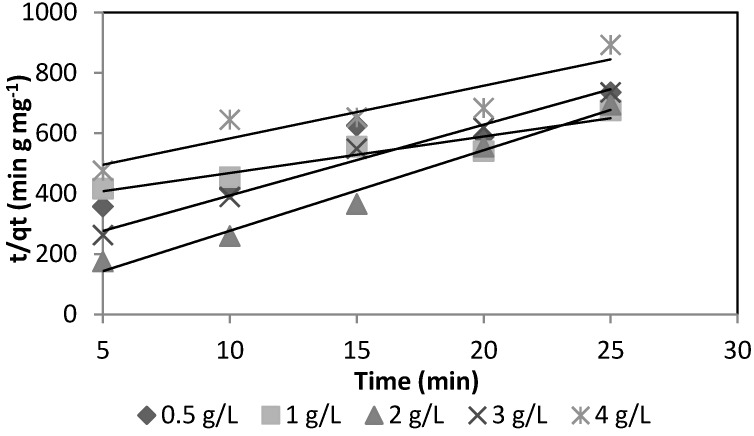
Kinetic sorption of As(V) for silica sand coated with Fe(III).

**Figure 6:**
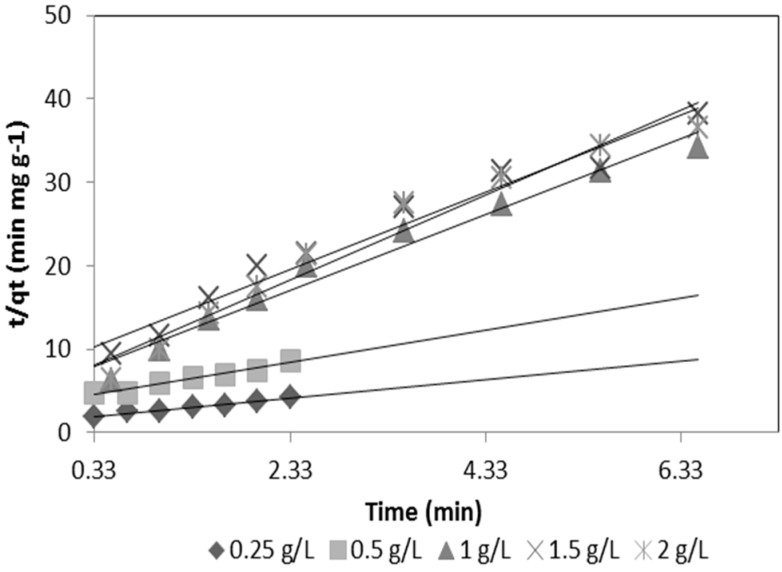
Kinetic sorption of As(V) for goethite.

Based on the design of the experiments, a statistically significant difference (*p* < 0.005) was found for the two adsorbent media for the initial concentration of arsenic, while a statistically significant difference (*p* < 0.005) for mass was shown only for goethite. The optimal conditions obtained for silica sand coated with Fe(III) were: As concentration of 0.392 mg·L^−1^, pH of 8.5 and mass of 4 g·L^−1^. Those for goethite were: As concentration of 0.360 mg·L^−1^, pH of 7.5 and mass of 2.0 g·L^−1^. The sorption kinetics for the two media is shown in Figure 5 and Figure 6.

To quantify the changes in sorption with respect to the time required by a suitable kinetic model, we use the pseudo-second-order equation:

(2) $$\frac{t}{q_{t}}=\frac{1}{k_{ad}{q_{e}}^{2}}+\frac{1}{q_{e}}t$$

where *q_t_* is the sorption capacity (mg·g^−1^), *q_e_* is the sorption capacity in equilibrium (mg·g^−1^), *k_ad_* is the rate constant of sorption (g·mg^−1^·min^−1^) and *h* is the initial sorption rate (mg·g^−1^·min^−1^) at t = 0:

(3) $$h=k_{ad}{q_{e}}^{2}$$

Figure 5 and Figure 6 show the As(V) kinetics sorption for silica sand coated with Fe(III) and goethite.

Table 5 shows that the rate constant of sorption (k_ad_) for goethite is 5.39 times greater than the k_ad_ for silica sand coated with Fe(III) and, therefore, synthesized goethite has a greater sorption capacity due to its larger surface area as compared to silica sand coated with Fe(III). It can be seen that the initial sorption rate (h) of goethite is higher than the silica sand coated with Fe(III). Studies conducted by Thirunavukkarasu *et al.* (2001; 2003) [6,14] and Paredes (2012) [9] determined k_ad_ values of 0.033 and 7.409 g·mg^−1^ min^−1^ for granular iron hydroxide and goethite, respectively.

**Table 5:** Pseudo-second-order kinetic sorption constants.

	Units	Silica Sand Coated with Fe (III)	Goethite
Mass	(g)	4	2
q_e_	mg·g^−1^	0.058	0.196
k_ad_	(g·mg^−1^·min^−1^)	0.745	4.019
h	(mg·g^−1^·min^−1^)	0.0024	0.155

**Figure 7:**
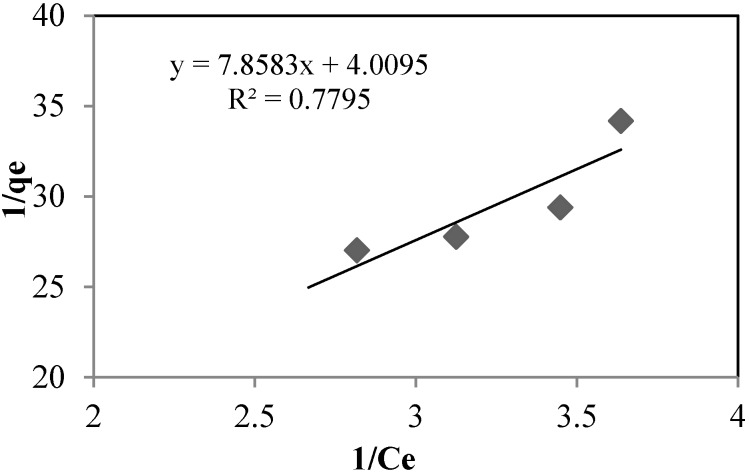
Langmuir (II) isotherm for silica sand coated with Fe(III). As_initial_: 0.392 mg·L^−1^. T: 22 °C.

**Figure 8:**
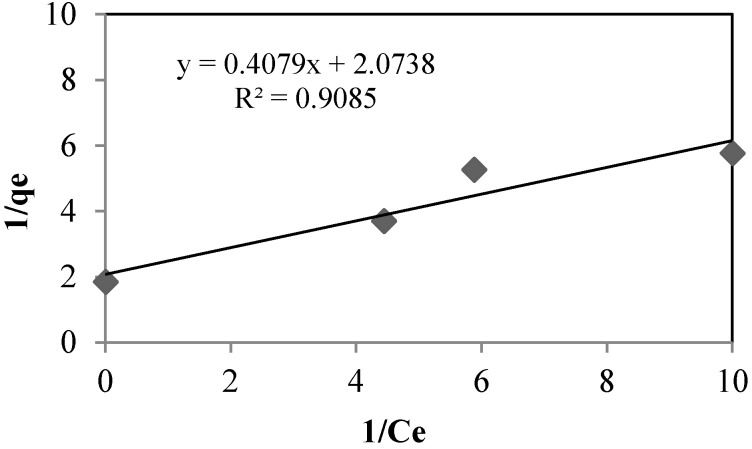
Langmuir (II) isotherm for goethite. As_initial_: 0.360 mg·L^−1^. T: 22 °C.

The sorption isotherms of arsenate using silica sand coated with Fe(III) and goethite at pH 8.5 and 7.5, respectively, are shown in Figure 7 and Figure 8, and the isotherm constants are shown in Table 6. For both, adsorption takes place according to the Langmuir model, type (II):

(4) $$q_{e}=\frac{q_{m}bC_{e}}{1+bC_{e}}$$

Langmuir (II)

(5) $$\frac{1}{q_{e}}=\frac{1}{q_{m}}+(\frac{1}{bq_{m}})+(\frac{1}{C_{e}})$$

where *q_e_* is the sorption capacity in the equilibrium (mg·g^−1^), *q_m_* is the maximum sorption capacity (mg·g^−1^), *C_e_* is the concentration in equilibrium (mg·L^−1^) and *b* is the constant related with the energy.

**Table 6:** Langmuir (II) isotherm constants.

	Units	Silica Sand Coated with Fe (III)	Goethite
q_m_	(mg·g^−1^)	0.2494	0.4822
b	-	0.5102	5.0841
*R*^2^	-	0.7795	0.9085

A comparison of the removal capacities of the selected sorbent materials towards As(V) is given in Table 7. The As(V) uptake determined in this work was higher than: iron oxide–coated sand (IOCS) ferrihydrite (FH) for goethite and granular ferric hydroxide (GFH). The goethite adsorbent seems to be a good alternative for the removal of As(V) species from an aqueous systems.

**Table 7:** A comparison of the produced materials’ As(V) sorption capacity (q_m_) with data from the literature, and the best fit Langmuir isotherm model.

Adsorbent	C_o_ (mg·L^−1^)	pH	q_m_ (mg·g^−1^)	Reference
Silica sand coated with Fe(III)	0.392	8.5	0.2494	This study
Goethite synthesized	0.360	7.5	0.4822	This study
Natural goethite	0.220	7.5	9.30	[15]
Iron oxide coated sand (IOCS)	0.325	7.4	0.183	[6]
Ferrihydrite (FH)	-	-	0.285	[6]
Crystalline hydrous ferric oxide (CHFO)	50	-	25	[16]
Granular ferric hydroxide (GFH)	0.50	7.6	0.159	[14]
Sulphuric acid acidified laterite (ALS)	0.1	7.0	0.923	[17]
Silica coated with Fe oxides (8%)	50	5.0	30.59	[17]
Silica coated with Al oxides (8%)	50	4.0	6.52	[17]

## 4. Conclusions

The adsorbent media with a more efficient removal of arsenic (98.61%) was goethite synthesized in the laboratory. The arsenic concentration obtained in the effluent was 0.005 mg/L, a value that complies with the Mexican norm NOM-127-SSA-1994 and WHO guidelines (2004). When fitting the experimental data to a pseudo-second-order equation, the kinetic sorption study shows goethite to have a high rate constant of sorption (k_ad_) as compared to other investigations. This is also seen for the maximum sorption capacity (q_m_) obtained by applying the Langmuir model type (II).
